# Preparation of a Cu(II)-PVA/PA6 Composite Nanofibrous Membrane for Enzyme Immobilization

**DOI:** 10.3390/ijms131012734

**Published:** 2012-10-05

**Authors:** Quan Feng, Bin Tang, Qufu Wei, Dayin Hou, Songmei Bi, Anfang Wei

**Affiliations:** 1 Key Laboratory of Eco-Textiles (Ministry of Education), Jiangnan University, Wuxi 214122, China; E-Mails: fengquan@ahpu.edu.cn (Q.F.); wanfang2564@yahoo.com.cn (A.W.); 2 Textiles and Clothing Department, Anhui Polytechnic University, Wuhu 241000, China; E-Mails: tangbin@ahpu.edu.cn (B.T.); houdayin0141@sina.com (D.H.); bisongmei18@yahoo.com.cn (S.B.)

**Keywords:** PVA/PA6, nanofibrous membranes, metal chelation, enzyme, immobilization

## Abstract

PVA/PA6 composite nanofibers were formed by electrospinning. Cu(II)-PVA/PA6 metal chelated nanofibers, prepared by the reaction between PVA/PA6 composite nanofibers and Cu^2+^ solution, were used as the support for catalase immobilization. The result of the experiments showed that PVA/PA6 composite nanofibers had an excellent chelation capacity for Cu^2+^ ions, and the structures of nanofibers were stable during the reaction with Cu^2+^ solution. The adsorption of Cu(II) onto PVA/PA6 composite nanofibers was studied by the Langmuir isothermal adsorption model. The maximum amount of coordinated Cu(II) (*q_m_*) was 3.731 mmol/g (dry fiber), and the binding constant (*K_l_*) was 0.0593 L/mmol. Kinetic parameters were analyzed for both immobilized and free catalases. The value of *V_max_* (3774 μmol/mg·min) for the immobilized catalases was smaller than that of the free catalases (4878 μmol/mg·min), while the *K_m_* for the immobilized catalases was larger. The immobilized catalases showed better resistance to pH and temperature than that of free form, and the storage stabilities, reusability of immobilized catalases were significantly improved. The half-lives of free and immobilized catalases were 8 days and 24 days, respectively.

## 1. Introduction

Enzymes are well known green catalysts which are highly specific and efficient. However, the applications of enzymes suffer from various problems, e.g., non-reusability, high-cost and instability [1]. Enzyme immobilization has become an effective way to overcome these limitations to some extent. The enzyme immobilized on the surface of insoluble supports can be recycled much more easily than a soluble enzyme. On the other hand, the multiple-point attachment to the support can restrict the undesirable conformational changes of enzyme proteins in practical applications, and maintain the activity of immobilized enzymes as much as possible [2–4].

Immobilization of enzymes can be achieved through physical adsorption on solid supports, micro-encapsulation, chelation bonds, covalent bonds or matrix entrapment. Among these, chelation bond seems to have some advantages over the other methods such as simplicity of the process, more stability and less possibility of inactivation of the immobilized enzyme [5].

Most of the transition metal ions such as Cu^2+^, Fe^3+^, Ni^2+^, Zn^2+^ can form stable complexes with electron-rich compounds and coordinate molecules containing O, N and S, for example, amine (NH_2_), hydroxyl (OH) and thiol (SH) groups.

Catalases are enzymes that decompose hydrogen peroxide (H_2_O_2_) into water and oxygen, which are commonly used in various fields, including the food, textile, agriculture and detergent and so on. The use of catalases is very effective in the aspects of lower resource utilization and energy consumption [6]. Every catalase protein consists of four subunits, and each of them includes a ferriporphyrin as a prosthetic group. Immobilization of enzymes on a metal chelated matrix is based on chelation bonds between the chelated metal ions on the support and active sites such as indole groups of tryptophanes, imidazole groups of histidines and thiol groups of cysteines [7].

Application of nanofibrous membranes for enzyme immobilization is becoming popular because of their large surface area. Nanofibrous membranes can improve the binding capacity of immobilized enzymes and increase the mass transfer kinetics when biocatalytic reactions occur on the surface of the nanofibrous membrane [8–10]. Electrospinning is the most effective technique for preparing nanofibrous membranes, in which a polymer jet is ejected when the electrostatic force applied to the polymer liquid, which overcomes the surface tension of the polymer solution. The jet is elongated and accelerated in the electrostatic field, followed by solvent evaporation and deposition on a substrate at random [11,12].

Poly (vinyl alcohol) (PVA) is a cheap, nontoxic, hydrophilic, biocompatible synthetic polymer, and there are plenty of hydroxy (OH) groups in it. PVA has been widely used in cell and enzyme immobilization [13]. In our previous work, PVA nanofibers were formed by electrospinning, and metal chelated nanofibers were prepared by reaction with metal ions solution, but it was found that PVA nanofibers had undergone a swelling phenomenon in aqueous solution, and the nanofibrous structures were distorted to a certain extent. In addition, we found that PVA/PA6 composite nanofibers could be formed by electrospinning, and PVA/PA6 composite nanofibers showed excellent structural stability in aqueous solutions of metal ions [14].

In this work, PVA/PA6 composite nanofibrous membranes were formed by electrospinning. Then, Cu(II) ions were incorporated into the hydroxyl groups and carbonyl groups of the nanofibers via metal chelation, and the chelated reaction kinetics of PVA/PA6 composite nanofibers to Cu(II) were established. Likewise, catalases were immobilized on the surface of the Cu(II)-PVA/PA6 composited nanofibrous membrane by chelated groups, and the activity and stability of the immobilized catalases were studied by examining the decomposition of H_2_O_2_. At the same time, the kinetic parameters of free and immobilized catalases were studied.

## 2. Results and Discussion

### 2.1. Adsorption Isotherm of Cu(II) on PVA/PA6 Composite Nanofibrous Membrane

In order to get the best values of the binding parameters from the experimental results, Equation 1 can be rearranged as below:

(1) $$\frac{1}{q}=\frac{1}{q_{m}K_{l}C_{e}}+\frac{1}{q_{m}}$$

**Figure 1:**
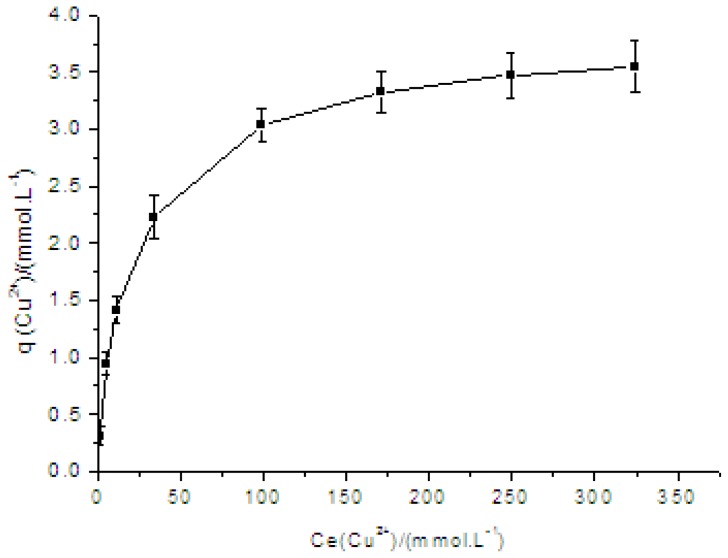
Adsorption isotherm of Cu^2+^ on the PVA/PA6 composite nanofibers. Bars represented standard deviations (*n* = 3).

**Figure 2:**
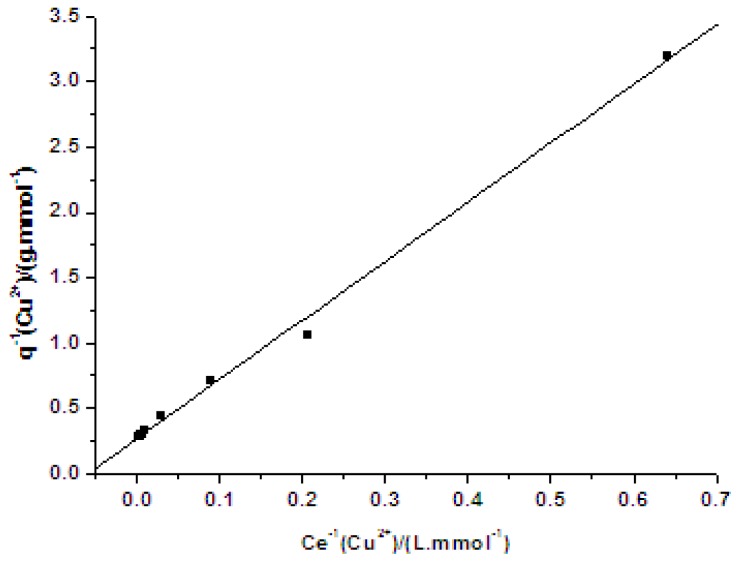
Relation of 1/*q* and 1/*C_e_*.

According to experimental results, plots of *q vs.* Ce and 1/*q vs.* 1/Ce are shown in Figures 1 and 2.

It is clearly observed that the adsorption of Cu(II) increased significantly with the rising concentration from 1.88 mmol/L to 327.79 mmol/L and gradually leveled off. The initial increase of Cu(II) adsorption might be due to many available chelating hydroxyl groups and carbonyl groups in PVA/PA6 composite nanofibers, then reached saturation gradually with the increase in the concentration of Cu(II).

The adsorption equilibrium data could be interpreted by the Langmuir absorption equation. The basic assumption of the Langmuir theory is once a metal ion occupies a reaction site, then no further adsorption occurs at the same location [15]. The reaction kinetics parameters could be calculated through straight line slope and ordinate intercept of plots. The reaction rate constant (*K_l_*) was 0.0593 L/mmol, and the maximum coordinate capacity (*q_m_*) of PVA/PA6 composite nanofibers to Cu(II) was 3.731 mmol/g (dry fiber). Comparing with our previous work, PVA/PA6 composite nanofibers showed obviously higher adsorption capability for Cu^2+^ ions than that of PVA nanofibers [2].

### 2.2. FTIR Spectra of Nanofibers

**Figure 3:**
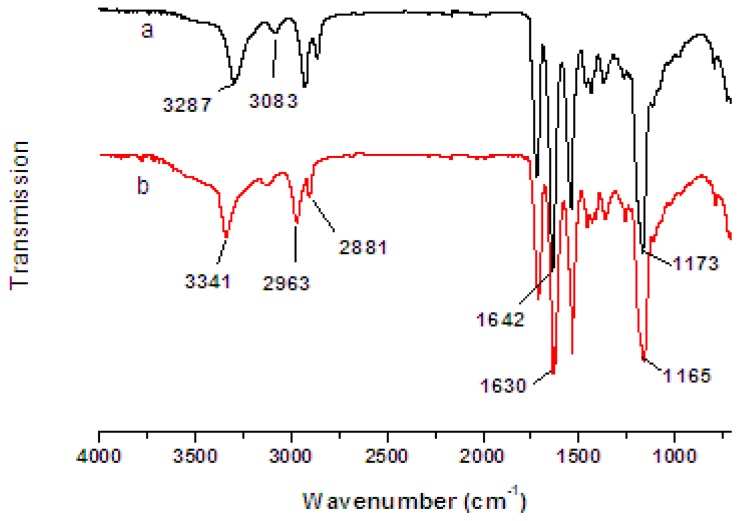
FTIR spectra of (**a**) PVA/PA6 composite nanofibers and (**b**) Cu(II)-PVA/PA6 metal chelated nanofibers.

The FTIR spectra of the PVA/PA6 composite nanofibers and the Cu(II)-PVA/PA6 metal chelated nanofibers are presented in Figure 3. The FTIR spectrum of the PVA/PA6 (Figure 3a) showed the following characteristic peaks: the bands of 3500 cm^−1^–2800 cm^−1^ correspond to the N–H, O–H and C–H stretching vibrations. The band at 1173 cm^−1^ was assigned to the C–O stretching vibration in PVA, and the band at 1642 cm^−1^ indicated the C=O stretching vibration in PA6.

The C-O band at 1173 cm^−1^ (Figure 3a) moved to 1165 cm^−1^ (Figure 3b), the C=O band at 1642 cm^−1^ (Figure 3a) moved to 1630 cm^−1^ (Figure 3b) due to the decrease of bond force constant coupled with decrease of the electron density, which indicated that the corresponding groups of PVA/PA6 coordinated with Cu(II). Moreover, the wide band of 3,287 cm^−1^ (Figure 3a) corresponding to the O–H stretching vibration moved to 3,341 cm^−1^ (Figure 3b) due to the increase of the relative mass of hydroxyl group, which was caused by the coordination of metal ions.

### 2.3. The Surface Morphologies of Nanofibrous Membrane

**Figure 4:**
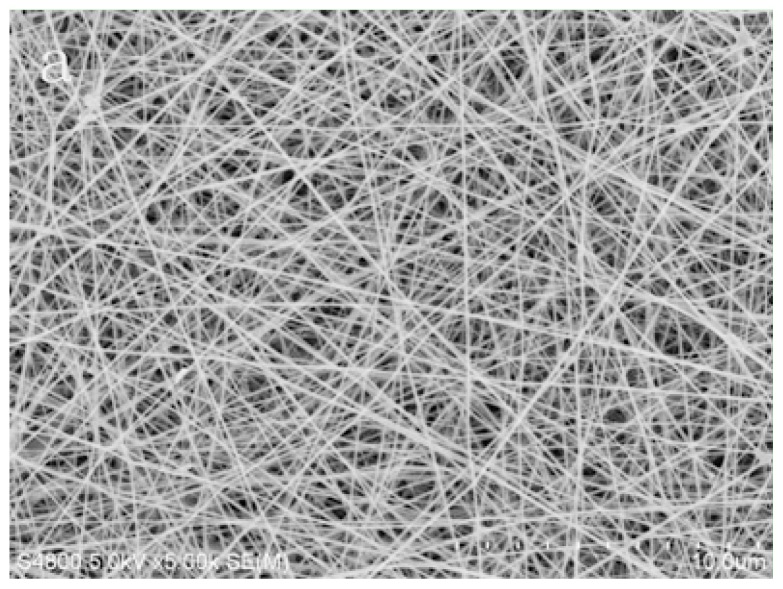

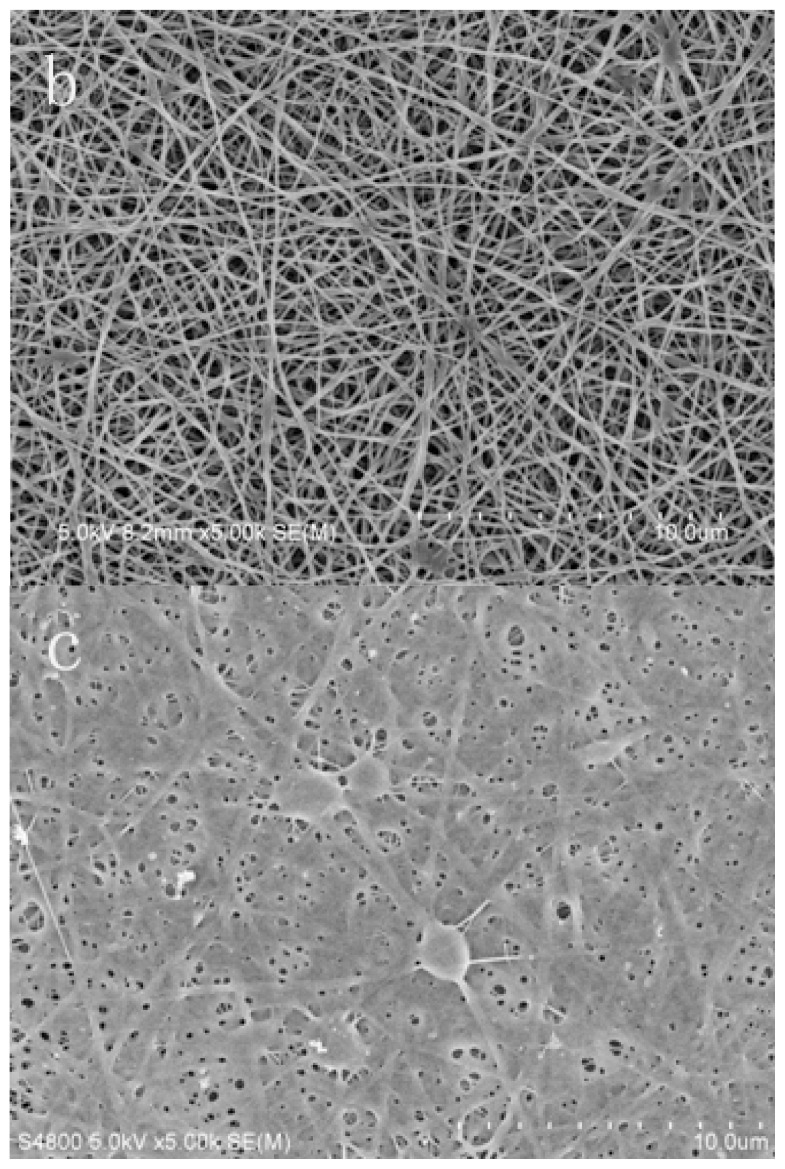
Micrographs of (**a**) original PVA/PA6 composite nanofibrous membrane; (**b**) and (**c**) PVA/PA6 composite nanofibers and PVA nanofibers after reaction with aqueous Cu^2+^ ions solution for 24 h, respectively.

The SEM images of the original PVA/PA6 composite nanofibrous membranes are displayed in Figure 4a. The electrospun PVA/PA6 composite nanofibrous membranes formed a fibrous membrane with random orientations. The nanofibrous membranes looked very even and the average diameter of the electrospun nanofibers ranged between 90 nm and 110 nm. The diameter of composite nanofibrous membranes did not change substantially after the reaction with aqueous solution of Cu(II) ions for 24 h, and the fibrous structures was not obviously distorted, as revealed in Figure 4b. However, the constructions of PVA nanofibrous membranes showed obvious damages because of swelling after the reaction with aqueous solution of Cu(II) ions for 24 h. The stability of nanofibrous membranes was obviously improved when PA6 was added to the composite, and the nanofibrous structure of PVA/PA6 composite nanofibers was still maintained in the metal ions aqueous solution.

### 2.4. Kinetic Parameters of Immobilized and Free Catalases

Maximum reaction rate *V_max_* and Michaelis-Menten constants *K_m_* are shown in Table 1. *K_m_* of the immobilized catalases was higher than that of free catalases, and *V_max_* of the immobilized catalases was smaller than that of free catalases.

These data indicated that affinity between immobilized catalases and substrate decreased comparing with free catalases. This might be affected by the structural changes of immobilized catalases, with less accessibility between substrate and active points of immobilized catalases caused by the space barriers presented by the supports [16,17].

### 2.5. Effect of pH and Temperature on the Enzyme Activity

**Figure 5:**
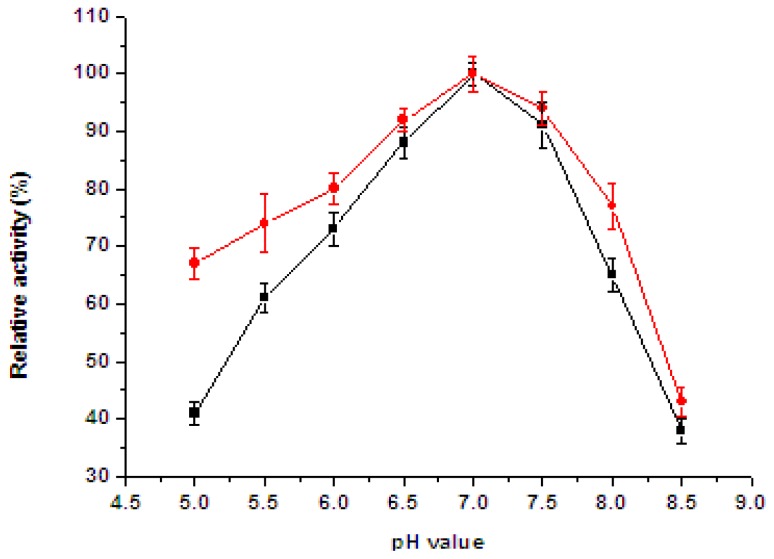
Effect of pH on the (●) immobilized and (■) free catalase. Bars represent standard deviations (*n* = 3).

The effect of pH on the activity of free and immobilized catalase is shown in Figure 5.

**Figure 6:**
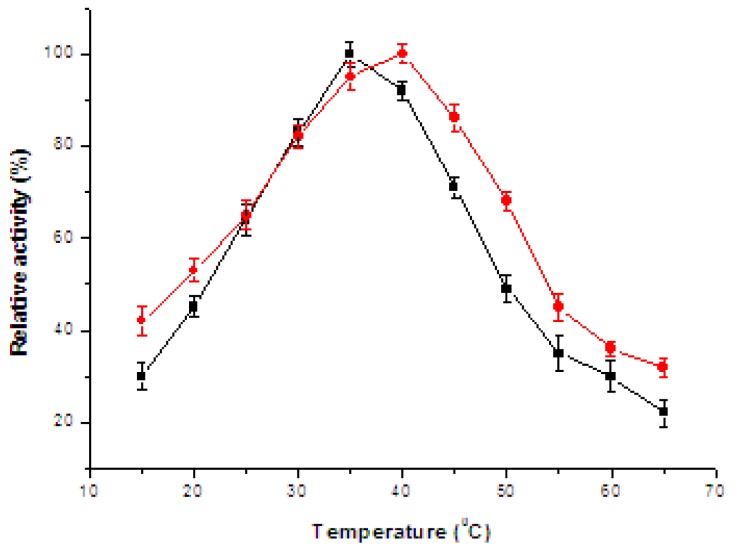
Effect of temperature on the (●) immobilized and (■) free catalases. Bars represent standard deviations (*n* = 3).

According to the experimental results, no significant shift of the optimal pH was observed. The optimal pH value was about 7.0 for free and immobilized enzyme, but the residual relative activity of the immobilized catalase was higher than that of the free one in the pH range between 5.0 and 8.5. It was found that the immobilized enzyme showed less sensitivity to pH than that of the free enzyme, probably because of the production of oxygen, forming foams, and causing slight diffusion limitation on the surface of nanofibrous membrane [18]. The effect of temperature on the activity of free and immobilized enzyme is depicted in Figure 6.

It is obvious that the initial relative activity was increased with the increase of temperature and decreased while the temperature was further increased for immobilized and free catalases. The optimum temperatures for the free and immobilized catalases were observed to lay at approximately 35 °C and 40 °C, respectively. At higher temperature range, the immobilized catalase exhibited higher stability than the free one, and it showed the anti-thermal properties of the immobilized enzyme. The multipoint interactions between enzyme and Cu(II)-PVA/PA6 support might reduce the degree of freedom of the spatial structure of enzyme, protecting it from deactivation at high temperatures.

### 2.6. Storage Stability and Reusability

**Figure 7:**
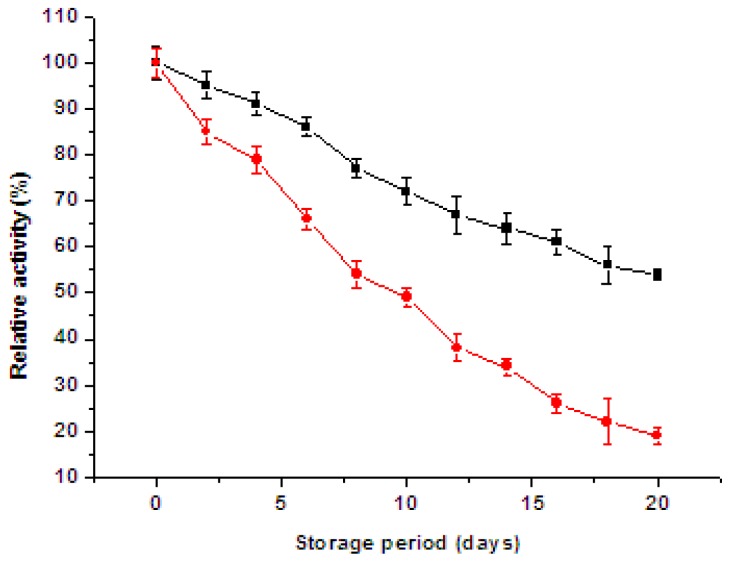
Storage stability of (■) immobilized catalases and (●) free catalases. Bars represent standard deviations (*n* = 3).

The storage stability of immobilized and free enzyme is presented in Figure 7. The residual activity of immobilized and free catalase was 54% and 19% after 20 days, and the half-lives of free and immobilized catalase were 8 days and 24 days, respectively. The results indicated that the storage stability of immobilized catalases was better than that of free catalases, which might be attributed to the immobilization of enzyme to a matrix. The immobilization of enzyme could limit the freedom of conformational changes, resulting in increasing stability towards denaturalization [19].

**Figure 8:**
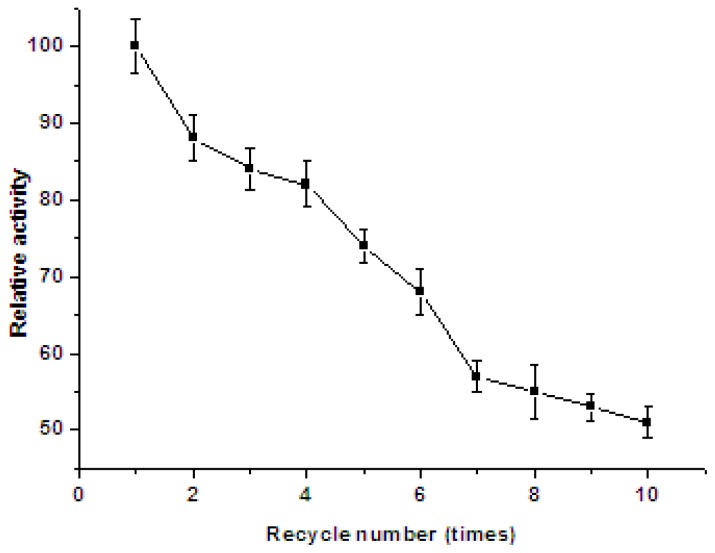
Reuse stability of the immobilized catalases. Bars represent standard deviations (*n* = 3).

According to the effect of repeated use on activity of immobilized catalase (Figure 8), after five repeated uses, immobilized catalases retained about 74% of their initial activity, and the residual activity of the immobilized enzyme was about 51% of the initial activity after 10 repeated uses, which showed its potential value in actual applications.

## 3. Materials and Methods

### 3.1. Materials

PA6 (weight-average molecular was 1800, characteristic viscosity was 2.8) and PVA (weight-average molecular was 84,000–89,000) obtained from Shanghai Kanghu Chemical (Shanghai, China). Bovine liver catalase (hydrogen peroxide oxidoreduction; EC. 1.11.1.6) were purchased from Sigma (Shanghai, China). Copper dichloride, hydrogen peroxide, formic acid and Coomassie Brilliant Blue (G250) for the Bradford protein assay were purchased from Shenyang Sinopharm Chemical Reagent (city, China). Hydrogen peroxide (30%) and the ingredients of phosphate buffer solution (PBS) such as NaCl, KCl, KH_2_PO_4_, K_2_HPO_4_ were analytical grade and used as received. Water used in all experiments was de-ionized.

### 3.2. Electrospinning

Electrospinning was carried out to fabricate PVA/PA6 composite nanofibers. PVA and PA6 were dissolved in formic acid (of 88% purity) at room temperature with stirring. The mass ratios of PVA:PA6 were 4%:12% in blended solutions. PVA/PA6 blended solutions were placed in a syringe (content of 20 mL) equipped with a 0.7 mm diameter spinner jet, and the solution flow rate was controlled by a microinfusion pump (WZ-50C2, Zhejiang, China). The high-voltage supplier (DW-P503-4AC, Tianjin, China) was used to connect the metal needles and the grounded collector to form the electrostatic fields. The blended polymer solutions were electrospun at the positive voltage of 15 kV with a collecting distance of 14 cm (the distance between the syringe needle tip and the collection roller covered with the aluminum foil). Nanofibers were collected by the roller for 24 h. On the other hand, PVA nanofibers were also prepared with the same parameters (weight ratio of PVA in solution was 8%) for comparison.

### 3.3. Chelation of Cu(II)

PVA/PA6 composite nanofibers (0.05 g) were put in flasks, each of them containing various concentrations of copper dichloride solution (50 mL). The flasks were stirred at 20 °C for 24 h. The nanofibrous membranes with adsorbed Cu(II) were washed with de-ionized water. The amount of adsorbed Cu(II) was calculated by using the concentrations of the Cu(II) in the initial solution and in the resulting solution. The Cu(II) concentration was determined by atomic absorption spectrophotometry (AAS). The Cu(II)-PVA/PA6 nanofibrous membranes were dried in a vacuum drying oven, at a temperature of 40 °C for 24 h. The reaction kinetics parameters were studied by the Langmuir isothermal adsorption model:

(2) $$q=\frac{q_{m}K_{l}C_{e}}{1+K_{l}C_{e}}$$

where *q_m_* is the maximum amount of the coordinated Cu(II), *K_l_* is the binding constant, *C_e_* is the equilibrium concentration. Fourier transform infrared spectroscopy (FTIR) spectra of the PVA/PA6 composite nanofibers and Cu(II)-PVA/PA6 nanofibers were recorded using a FTIR spectrometer (NICOLET NEXUS 470). Surface morphologies of Cu(II)-PVA/PA6 and Cu(II)-PVA nanofibers were examined using FE-SEM (S-4800). All samples were coated with the platinum by sputtering before scanning electron microscope observation.

### 3.4. Enzyme Immobilization

Cu(II)-PVA/PA6 chelated nanofibrous membranes (coordinated Cu^2+^ ions = 3.03 mmol/g) were immersed in 50 mL of catalase solution (0.3 mg/mL) for 4 h at 20 °C in shakers while stirring continuously. Catalases were dissolved in 50 mM PBS (value of pH was 7.0). Then, the nanofibrous membranes were removed from the solution and rinsed with the same PBS until no soluble protein was detectable. The method of Bradford was applied to determine the concentration of enzyme, the amount of the catalase was determined by spectrophotometry according to the absorbance of Coomassie Brilliant Blue and catalases at 595 nm, and the amount of the bound enzyme was calculated as:

(3) $$Q=\frac{(C_{0}-C)V}{m}$$

where *Q* is the amount of catalases bound onto unit mass of nanofibrous membranes (mg/g), *C_0_* and *C* are the initial and equilibrium enzyme concentrations in the solution (mg/mL), *V* is the volume of the catalases solution, and *m* is the mass of the nanofibrous membranes.

### 3.5. Activity Assays

The activity of the free and immobilized catalases was determined spectrophotometrically by the measurement of decrease in the absorbance change of hydrogen peroxide at 240 nm. Immobilized catalases were mixed with hydrogen peroxide solution (100 mL, 50 mmol/L). The reaction was kept at 35 °C for 3 min, and the specific activity of enzyme was calculated by the following formula:

(4) $$v=\frac{\Delta A\times V}{T\times K\times Ew}$$

where *v* is specific activity of enzyme and immobilized enzyme (μmol/mg·min), Δ*A* is the absorbance decrease of the solution at 240 nm, *V* is the volume of the hydrogen peroxide solution (mL), *T* is the time of reaction (min), *K* is the molar extinction coefficient of hydrogen peroxide at 240 nm (*K* = 0.033 L/mmol cm), and *E_w_* is the amount of enzyme (mg).

The catalytic mechanism of catalases is presented as follows [20]:

$$\begin{array}{l} \text{CAT }({\text{Por}-\text{Fe}}^{\text{III}})+{\text{H}}_{2}{\text{O}}_{2}\to \text{Cpd I}({\text{Por}}^{+}{-\text{Fe}}^{\text{IV}}=\text{O})+{\text{H}}_{2}\text{O}\\ \text{Cpd I}({\text{Por}}^{+}{-\text{Fe}}^{\text{IV}}=\text{O})+{\text{H}}_{2}{\text{O}}_{2}\to \text{CAT }({\text{Por}-\text{Fe}}^{\text{III}})+{\text{H}}_{2}\text{O}+{\text{O}}_{2} \end{array}$$

### 3.6. Determination of Kinetic Parameters

The effect of hydrogen peroxide concentration on the activity was tested. *V_max_* and *K_m_* values of free and immobilized catalases were calculated by the Lineweaver-Burk plots:

(5) $$\frac{1}{v}=\frac{K_{m}}{V_{\text{max}}}\times \frac{1}{\left[S\right]}+\frac{1}{V_{\text{max}}}$$

where *K*_m_ is the Michaelis constant, *v* and *V_max_* represent the initial and maximal rate of the reaction, [*S*] is the concentration of the hydrogen peroxide. Kinetics parameters for immobilized and free enzyme were investigated at 35 °C and the concentrations of hydrogen peroxide ranged from 20 to 200 mmol/L (pH 7.0).

### 3.7. Dependence of Temperature and Value of pH

The immobilized and free enzyme were mixed with hydrogen peroxide solution (100 mL, 50 mmol/L) respectively, at temperatures ranging from 15 °C to 65 °C. The optimum pH values of free and immobilized enzyme were investigated at 35 °C for 3 min. PBS (pH 5.0–8.5) was used for pH dependence study.

### 3.8. Storage Stability and Reusability

The stability of free and immobilized catalases at storage was measured by calculating their activity retention during 20 days at 4 °C in 50 mM PBS (pH 7.0), using 2 days intervals, then a sample was removed and determined for the enzyme activity as described above. The half-lives of free and immobilized catalases were calculated by the following formulas.

(6) $$\left[E_{t}]=[E_{0}]\;\text{exp}(-K_{d}t\right)$$

(7) $$t_{1/2}=\frac{\text{ln }2}{K_{d}}$$

where *K*_d_ is the decay constant, *E*_0_ is the initial activity of enzyme, *E*_t_ is the residual activity of enzyme, *t*_1/2_ is the half-life of enzyme. In addition, the reusability of immobilized catalases was studied. Immobilized catalases were reused 10 times within 10 days (once a day). After each reaction run, the enzyme was washed with PBS to remove any residual substrate, and the retention of activity of the immobilized enzyme was conducted under the optimum conditions.

## 4. Conclusions

Electrospinning was carried out to fabricate PVA/PA6 composite nanofibrous membranes, and Cu(II)-PVA/PA6 nanofibrous membranes were prepared for catalase immobilization. The storage stability was significantly improved and the reusability of immobilized catalases was very high after immobilization onto the Cu(II)-PVA/PA6 nanofibrous membranes. These results indicated that the PVA/PA6 composite nanofibers showed excellent structural stability in aqueous solution, and the immobilized catalases had a high affinity with the support, which demonstrated that the catalases immobilized on Cu(II)-PVA/PA6 nanofibrous membranes may have potential in various applications.

## Figures and Tables

**Table 1:** The amount of bound enzyme and the kinetic parameters of the immobilized and free enzyme.

	Amount of bound enzyme (mg/g fibers)	Specific activity (Units/mg)	*K_m_* (mM)	*V_max_* (μmol/mg·min)
Free catalase		3400	26.815	4878
Immobilized catalases	−64	2150	41.132	3774
